# Supplementary material for: Cyclosporine A inhibits MRTF‐SRF signaling through Na+/K+ ATPase inhibition and actin remodeling
Source: FASEB Bioadv. 2019 Aug 24;1(9):561–78. doi: 10.1096/fba.2019-00027 (PMC6996406; doi:10.1096/fba.2019-00027)
Supplement: Supplementary file 1 [file FBA2-1-561-s001.docx]

Figure S1: Dynamic mapping of LLC PK-1 proteome highlights CNI-specific expression profiles of actin family cytoskeletal proteins. A, STRING visualization of iTRAQ-monitored actin family cytoskeletal protein network (PANTHER Protein Class PC00041). B, Heat-map representation of the identified actin family cytoskeletal proteins. Cut-offs for biological significance were calculated as Mean ± 1 SD (1.02 ± 0.10) based on the approximation of the iTRAQ ratio frequency distribution. Lower cut-off: 0.92 (green); upper cut-off: 1.12 (red)

Figure S2: Inhibitors of the RhoGTPases pathway elicited CsA-like features and potentiated CsA effects. A, Quantification of red fluorescence-positive area. Mean ± SEM, One-way ANOVA plus Tukey’s post-test (p<0.01**,p<0.001***) (n=3). B, SRF transcription activity was measured by luciferase gene reporter assay in LLC PK-1 SRE. Mean ± SEM. One-sample t-test for versus control comparison, One-way ANOVA plus Tukey’s post-test for multiple condition comparison (p<0.01**,p<0.001***) (n=3). Drug condition: Vehicle (0.5% Ethanol-0,2% DMSO), 5 µM CsA, 10 µM Y27632, Y27632 + CsA, 10 µM EHT1864, EHT1864 + CsA. Exposure time: 24 h

Figure S3: Molecular modeling of CsA docking into Na^+^/K^+^-ATPase. A, Binding poses of CsA docking into Na^+^/K^+^-ATPase open conformation (E2 state). B, Binding poses of CsA docking into Na^+^/K^+^-ATPase closed conformation (E1 state). C, Close-up of site C-located binding poses of CsA docking into Na^+^/K^+^-ATPase closed conformation (E1 state)
