# Supplementary material for: Cyclosporine A inhibits MRTF‐SRF signaling through Na+/K+ ATPase inhibition and actin remodeling
Source: FASEB Bioadv. 2019 Aug 24;1(9):561–78. doi: 10.1096/fba.2019-00027 (PMC6996406; doi:10.1096/fba.2019-00027)
Supplement: Supplementary file 2 [file FBA2-1-561-s002.pdf]

A

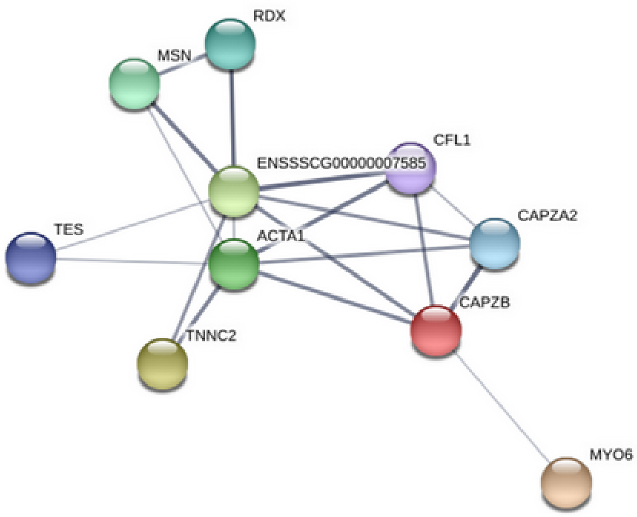

B

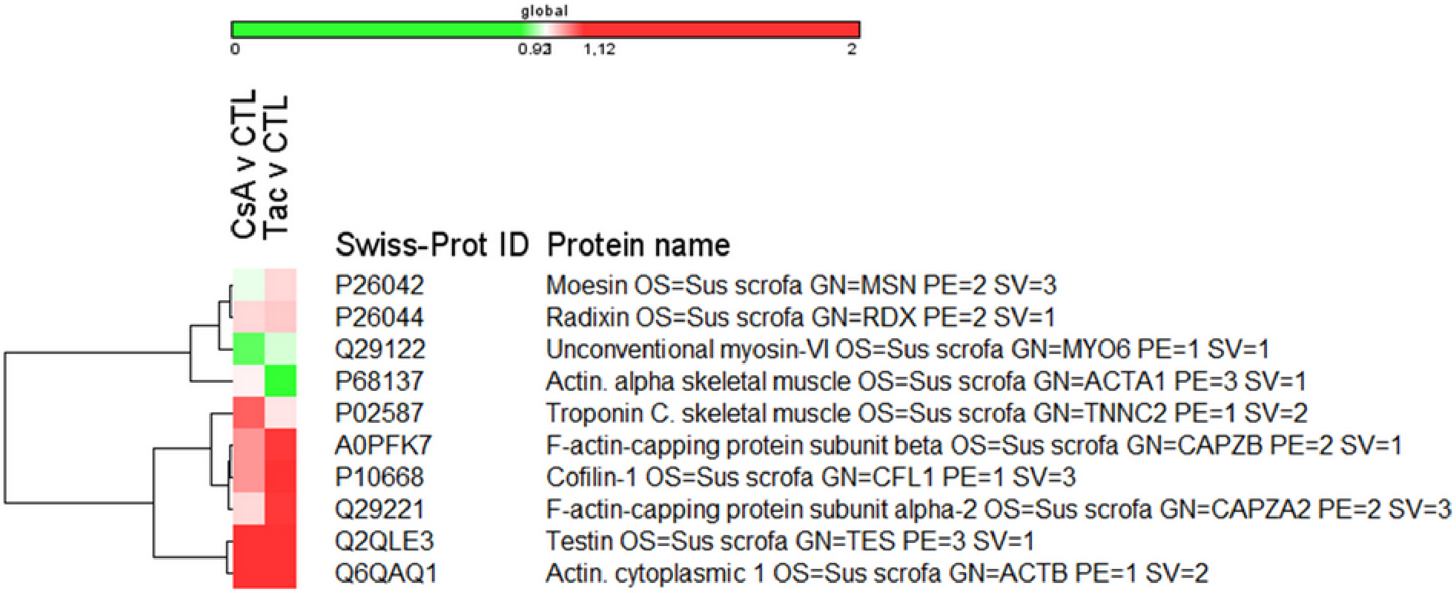

Figure EV1 – Dynamic mapping of LLC PK-1 proteome highlights CNI-specific expression profiles of Actin family cytoskeletal proteins.

A. STRING visualization of iTRAQ-monitored Actin family cytoskeletal protein network (PANTHER Protein Class PC00041)

B. Heat-map representation of the identified Actin family cytoskeletal proteins. Cut-offs for biological significance were calculated as Mean ± 1 SD (1.02 ± 0.10) based on the approximation of the iTRAQ ratio frequency distribution. Lower cut-off: 0.92 (green); upper cut-off: 1.12 (red).
