# Supplementary figures and images for: Cyclosporine A inhibits MRTF‐SRF signaling through Na+/K+ ATPase inhibition and actin remodeling
Source: FASEB Bioadv. 2019 Aug 24;1(9):561–78. doi: 10.1096/fba.2019-00027 (PMC6996406; doi:10.1096/fba.2019-00027)

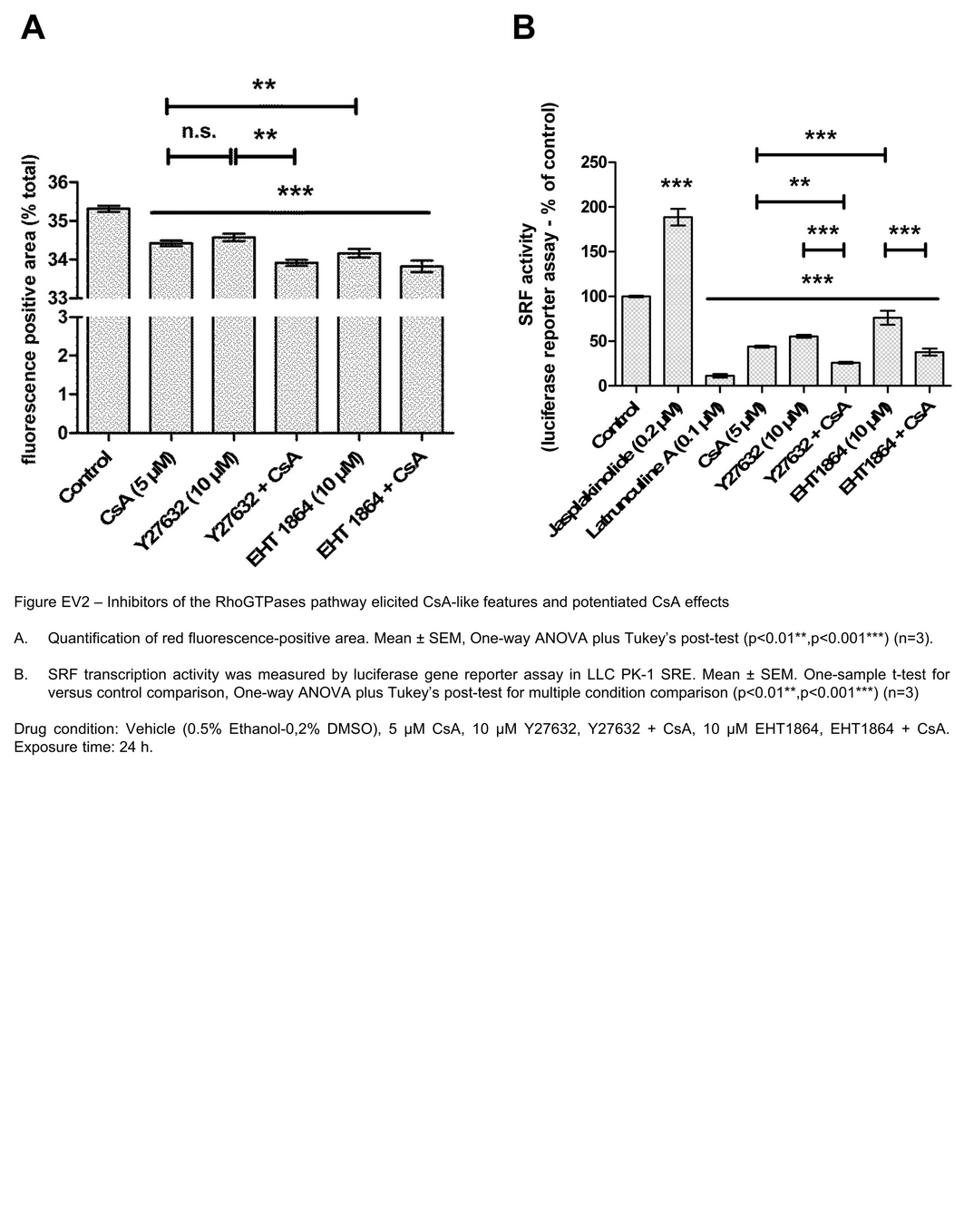

Supplement: Supplementary file 3 [file FBA2-1-561-s003.tif]
