# Supplementary material for: Cyclosporine A inhibits MRTF‐SRF signaling through Na+/K+ ATPase inhibition and actin remodeling
Source: FASEB Bioadv. 2019 Aug 24;1(9):561–78. doi: 10.1096/fba.2019-00027 (PMC6996406; doi:10.1096/fba.2019-00027)
Supplement: Supplementary file 4 [file FBA2-1-561-s004.pdf]

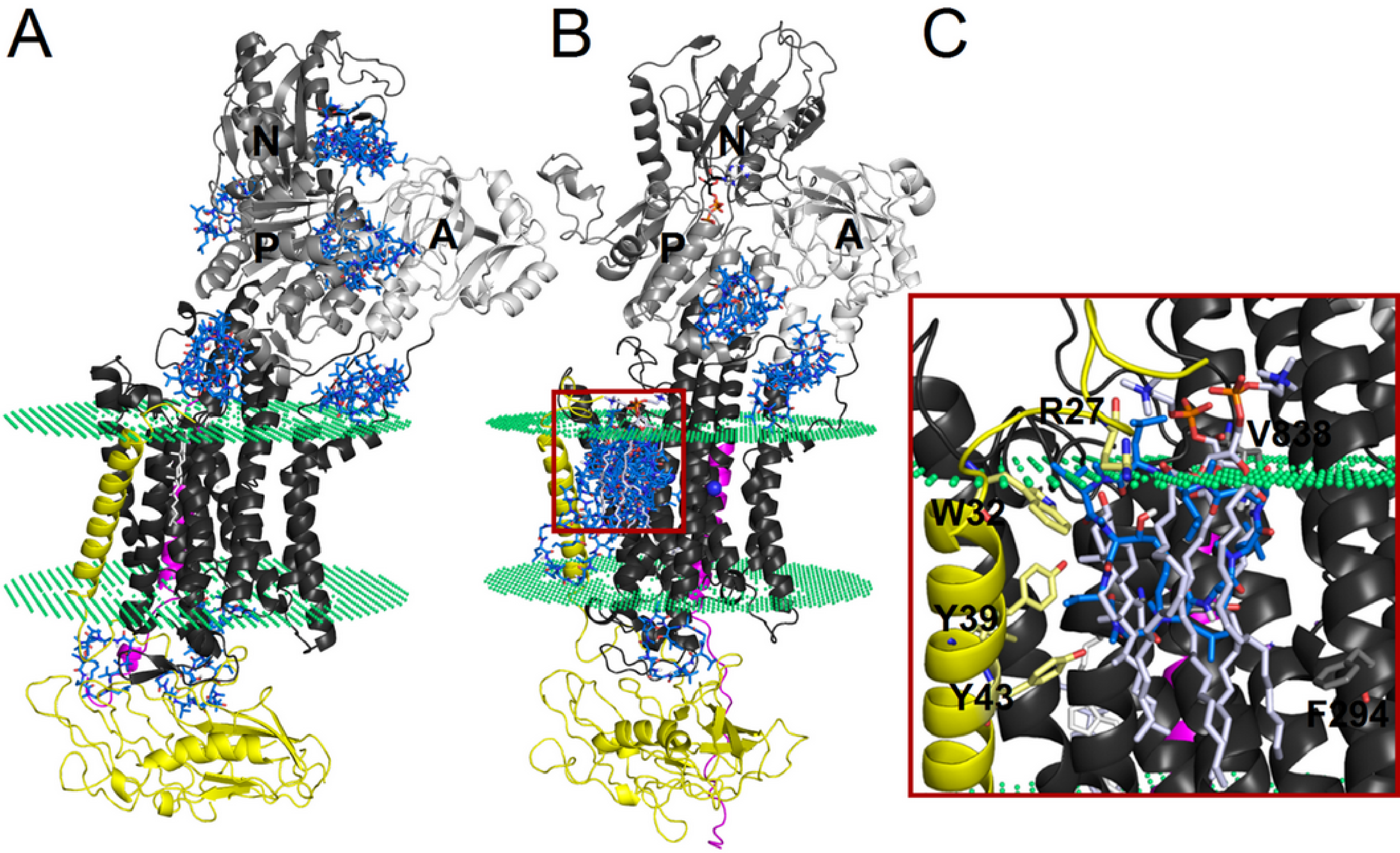

Figure EV3 – Molecular modelling of CsA docking into Na⁺/K⁺-ATPase

A. Binding poses of CsA docking into Na⁺/K⁺-ATPase open conformation (E2 state)
B. Binding poses of CsA docking into Na⁺/K⁺-ATPase closed conformation (E1 state)
C. Close-up of site C-located binding poses of CsA docking into Na⁺/K⁺-ATPase closed conformation (E1 state)
